# A guide to simple, direct, and quantitative *in vitro* binding assays

**DOI:** 10.14440/jbm.2017.161

**Published:** 2017-01-20

**Authors:** Stefanie Lapetina, Hava Gil-Henn

**Affiliations:** Faculty of Medicine in the Galilee, Bar-Ilan University, Safed 1311520, Israel

**Keywords:** *in vitro*, binding, protein-protein interaction, pull-down, quantification

## Abstract

Recent advances in proteomic screening approaches have led to the isolation of a wide variety of binding partners to interacting proteins and opened an avenue to analyze and understand signaling pathways. The study of protein-protein interactions is a key component in elucidating and understanding signaling pathways. Despite the importance of these interactions, very few studies are quantitative or report binding affinities. Here we present a simple method for examination and analysis of direct protein-protein binding interactions between two purified proteins. In the quantitative pull-down assay, one protein (the bait protein) is immobilized on beads whereas a second protein (the prey) is kept in solution. The concentration of the bait protein is kept constant, whereas the concentration of the prey protein is increased until binding saturation is achieved. After incubation, the beads are precipitated to separate unbound prey protein in solution from prey protein bound to the bait. The fraction of bound prey protein can then be loaded on a protein gel and the resulting bands can be analyzed with standard software. The quantitative pull-down assay with purified recombinant proteins provides a simple method to obtain dissociation constants (*K*_d_). These quantifications are invaluable to compare relative binding of proteins, to map binding sites, and to show that binding is direct. This assay presents a powerful method to quantitatively analyze protein-protein interactions with tools that are available in most biochemistry laboratories and does not require the use of specialized or expensive equipment.

## BACKGROUND

Every aspect of cellular function within an organism, including proliferation and survival, metabolism, cytoskeletal organization and gene transcription, is executed by signal transduction [1]. The process of signal transduction is dependent on specific protein-protein interactions that are mediated by modular protein domains that confer specific binding activity to the proteins in which they are found. Recent advances in high throughput proteomic screens to identify binding partners for interacting proteins and the availability of high-resolution three-dimensional structures of such interacting proteins has opened an avenue for analyzing and understanding signaling pathways [2]. One of the efficient ways for analyzing signaling pathways is by exploring direct protein-protein interactions between pathway participants. While several *in vitro* methods for testing direct binding of two proteins exist, many binding experiments are poorly designed and fall short of extracting all of the useful information available from the valuable reagents that were collected to do the experiment. In particular, many experiments fail to measure the affinity of the reactants to each other. Because binding reactions vary in strength, the answer to whether two molecules interact with each other should always be quantitative with a number that describes the affinity [3].

**Figure 1.**
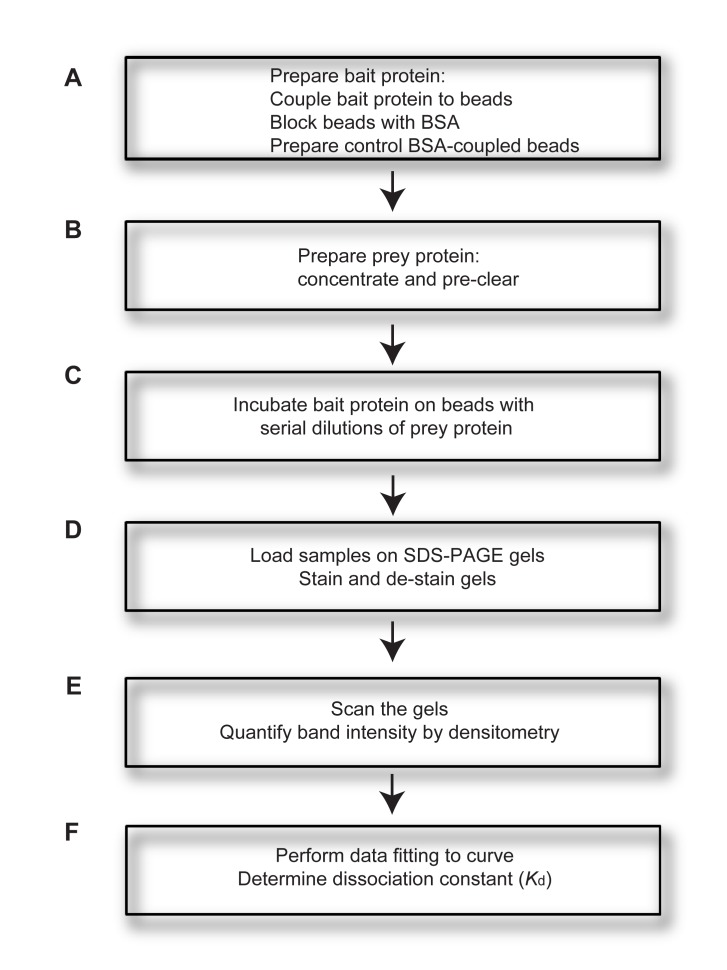
**Workflow diagram of the in vitro quantitative pull-down assay. A.** Purified bait protein is coupled to beads and beads are blocked with BSA to prevent non-specific binding. As a control, BSA-coupled beads are prepared. **B.** Purified prey protein is prepared by concentration and pre-clearing, to avoid non-specific binding to bait-conjugated beads. **C.** Bait protein is incubated with serial dilutions of prey protein to allow binding. **D.** Samples are loaded on SDS-PAGE gels, followed by staining and de-staining of gels. **E.** Gels are scanned and band intensities are quantified by densitometry. **F.** Data fitting to curve is performed and the dissociation constant (*K*_d_) is calculated.

Here we present a simple method for examination and analysis of the direct protein-protein binding interaction between two purified proteins. In the quantitative pull-down assay, one protein (the bait protein) is immobilized on beads whereas a second protein (the prey) is kept in solution. This can be achieved by several methods, such as affinity tags, antibody immobilization, or covalently linking the bait protein to the beads. The concentration of the bait protein is kept constant, whereas the concentration of the prey protein is increased until binding saturation is achieved. Most commonly, a constant concentration of protein-bound beads is incubated with increasing concentrations of the prey protein in solution. After incubation, the beads are spun down to separate unbound prey protein in solution from prey protein bound to the bait. Protein-protein interactions are then disturbed by methods depending on the initial immobilization of the bait protein (ranging from the addition of competitive reagents, pH changes, or boiling of the beads). The fraction of bound prey protein can then be loaded on a protein gel and the resulting bands can be analyzed with standard software (**Fig. 1**).

The *in vitro* pull-down assay shows that the interactions of two proteins are direct and are not facilitated by the presence of other proteins or additional macromolecules. When done correctly, this assay can be used to calculate the affinity of binding of the two proteins, usually represented by the dissociation constant, *K*_d_. This allows us to compare binding affinities of different proteins to a given binding partner. We can then map the binding sites by introducing truncation or point mutations in either or both of the binding partners. A properly designed assay allows the researcher to map sites that are necessary and sufficient for binding between the two proteins. Quantitative pull-down assays are a powerful method to quantitatively analyze protein-protein interactions with tools that are available in most biochemistry laboratories, as they do not require the use of specialized or expensive equipment.

## MATERIALS

### Reagents

• AminoLink Plus coupling resin (Thermo Fischer Scientific, cat. # 20501)
• BupH phosphate buffered saline packs (Thermo Fischer Scientific, cat. # 28372)
• NaOH, 1M (Sigma-Aldrich, cat. #72082)
• Bradford dye (BioRad, cat. # 500-0006)
• Bovine Serum Albumin (BSA) (Sigma-Aldrich, cat. # 9048-46-8)
• HEPES (Sigma-Aldrich, cat. # 7365-45-9)
• Triton X-100 (Sigma-Aldrich, cat. # 9002-93-1)
• Glycerol (Sigma-Aldrich, cat. # G5516)
• Tris Base (Sigma-Aldrich, cat. # 77-86-1)
• NaCl (Sigma-Aldrich, cat. # S3014)
• Sodium dodecyl sulfate (SDS), 20% (w/v), (BioRad, cat. # 1610418)
• Coomassie brilliant blue G-250 (BioRad, cat. # 1610406)
• Methanol, anhydrous (Sigma-Aldrich, cat. # 322415)
• Ammonium Sulfate (Sigma-Aldrich, cat. # 7783-20-2)
• Phosphoric acid (Sigma-Aldrich, cat. # 7664-38-2)
• Bottle top filter, (Sigma-Aldrich, cat. # 430516)
• 2-Mercaptoethanol, (Sigma-Aldrich, cat. # 60-24-2)
• Bromophonol blue (BioRad, cat. # 1610404)
• Amicon Ultra 15 centrifugal filters (Millipore, cat. # UFC903008; cutoff depends on size of protein)

### Recipes

• Coupling buffer: 3.65 × phosphate buffered saline (PBS), pH 7.2, or at NaCl concentration in which the bait protein is stable (*i.e.* 3.65 × corresponds to 500 mM NaCl).
• Sodium cyanoborohydride, 5M: Dissolve 0.314 g of Sodium cyanoborohydride (NaCNBH3) into a final volume of 1 ml NaOH (1M).
• Quenching buffer (1 M Tris, pH 7.25): Dissolve 60.57 g of Tris base in 300 ml double distilled water (DDW). Adjust pH to 7.25. Adjust volume to 500 ml with DDW.
• Blocking buffer: Dissolve 1g of BSA in 10 ml of quenching buffer (1 M Tris, pH 7.25). Filter-sterilize and store at 4^°^C.
• Wash solution (1 M NaCl): Dissolve 29.22 g NaCl in 500 ml DDW for a final concentration of 1 M.
• Binding buffer: Dissolve 2.98 g of HEPES in 300 ml DDW. Adjust pH to 7.25. Add 2.92 g NaCl, 50 μl Triton X-100, and 25 ml Glycerol and adjust the volume to 500 ml with DDW. Final concentration is 25 mM HEPES, 100 mM NaCl, 0.01% Triton X-100, and 5% Glycerol, 1 mM DTT.
• Coomassie blue-silver stain [4]: Add 100 ml phosphoric acid to 300 ml DDW. Add 100 g ammonium sulfate, stir until dissolved. Slowly add 1.2 g Coomassie blue G-250. Stir until dissolved (this may take overnight). Add 200 ml methanol and DDW to a final volume of 1 L. Filter through a bottle-top filter.
• Laemmelli sample buffer (LSB), 4 ×: Add 4 ml of 20% SDS to 2 ml of 1M Tris, pH 6.8. Add 2 ml glycerol and 0.8 ml of β -mercaptoethanol. Add a small pinch of bromophenol blue. Adjust volume to 10 ml. Stir or rotate until ingredients are well combined. Store aliquots at −20°C.
**TIP**: adding less bromophenol blue yields a lighter blue LSB buffer. This will make it easier to distinguish the beads from the supernatant after boiling (Step 3.8 of the protocol below).

### Equipment

• End-over-end tube rotator (Argos Technologies, R2001)
• Eppendorf table-top centrifuge at 4°C (Eppendorf, 5417R)
• pH meter (Sartorius, PB-11)
• Spectrophotometer or NanoDrop (Thermo Scientific, NanoDrop 2000c)
• Dry bath incubator (Major Science, MD-01N-110/220)
• Protein electrophoresis system (Bio-Rad, cat. #1658004)
• Gel documentation system (Syngene, InGenius 3)

### Software

• ImageJ (National Institute of Health, USA: http://imagej.nih.gov/ij) or a similar quantification software.
• GraphPad Prism (La Jolla, California, USA: http://www.graphpad.com) or a similar data analysis software.

## PROCEDURE

**Figure 2.**
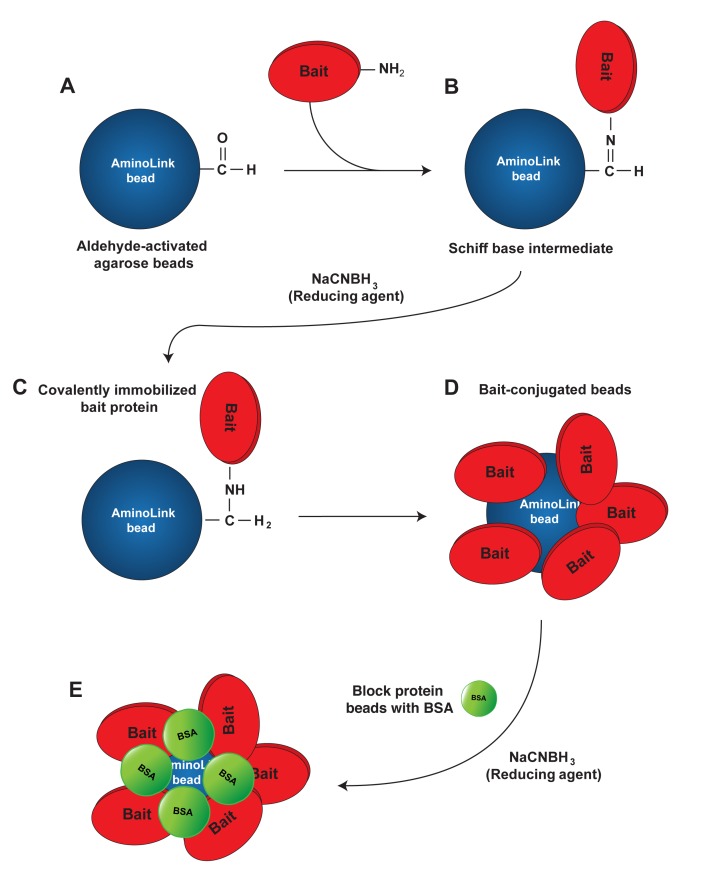
**Coupling the bait protein to AminoLink beads**. AminoLink beads are aldehyde-activated agarose beads (**A**) that react with primary amines in the N-terminus or at lysine residues of the bait protein through a Schiff base intermediate (**B**). Addition of a reducing agent (sodium cyanoborohydride, NaCNBH_3_) leads to the formation of a stable secondary amine bond and a covalently immobilized bait protein (**C**). After conjugating the beads to bait protein (**D**), the remaining active sites on the beads are blocked by the addition of BSA and reducing agent (**E**), to prevent non-specific binding of prey protein to the beads.

**Figure 3.**
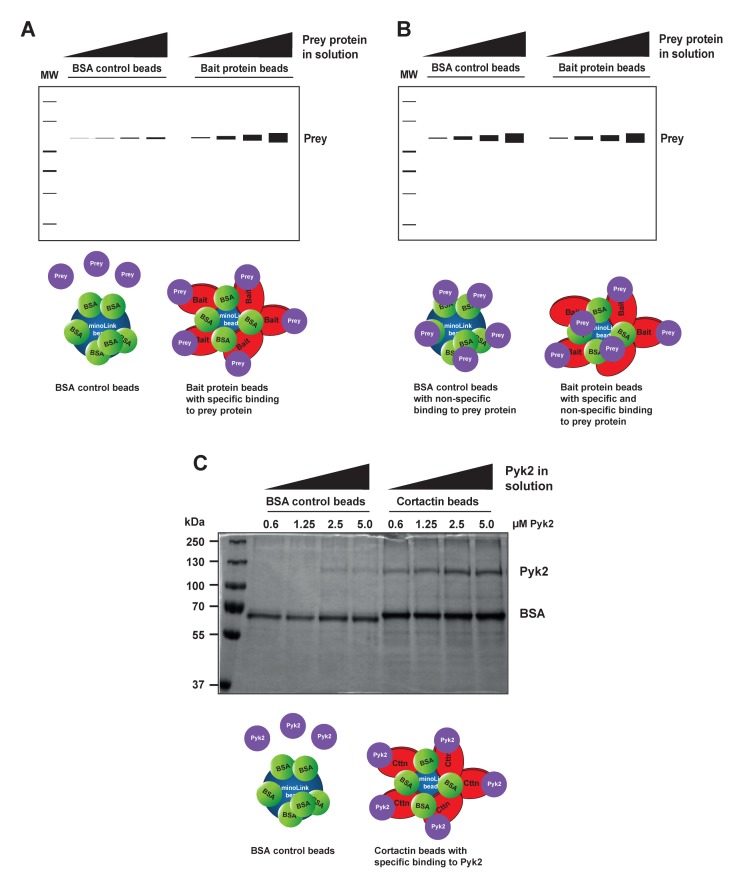
**Screening for binding conditions. A.** Graphic representations of a valid binding screen, in which no significant binding of prey protein to BSA control beads occurred. **B.** An example of a test experiment in which the prey protein nonspecifically bound to BSA control beads. **C.** An example of an actual screen in which increasing amounts of purified Pyk2 (prey) (0.6, 1.25, 2.5, and 5 μM) in solution were incubated with constant concentrations of purified cortactin (Cttn, bait) conjugated to AminoLink beads or BSA-conjugated beads as control. Note that only a negligible amount of Pyk2 was pulled-down with BSA control beads. Cartoons below gels depict either specific or non-specific binding of prey protein or Pyk2 to the protein beads. It is common for BSA to leak off the beads because high concentrations are used for blocking. This can be used as a convenient loading control, to ensure that equal amount of beads was used for all samples. If the prey protein has a molecular weight that is similar to BSA, beads can be blocked with ethanolamine instead.

**Figure 4.**
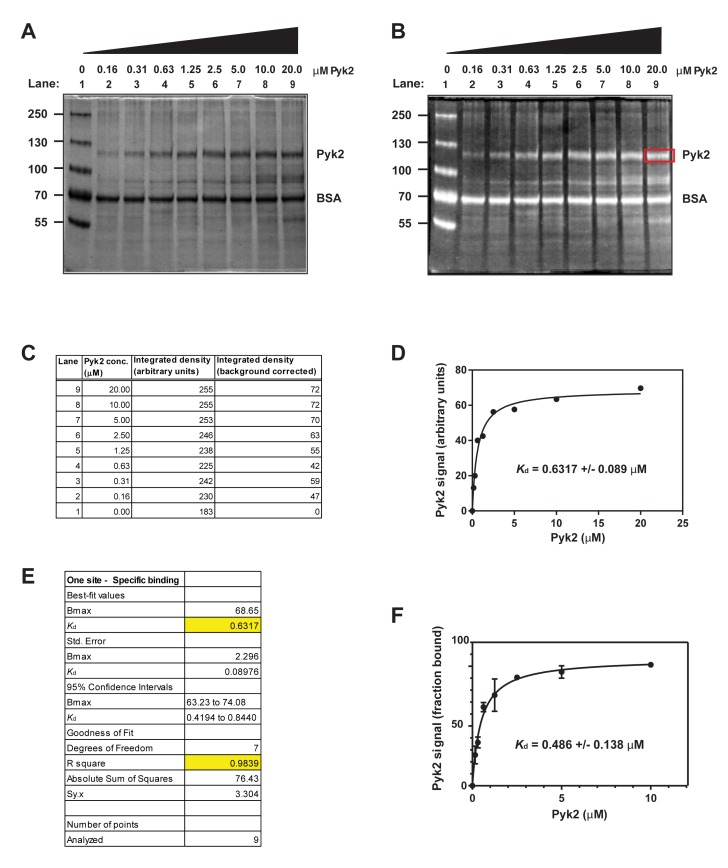
**A sample of binding curve and values for protein-protein binding interaction.** An example of SDS-PAGE gel from a quantitative pull-down experiment in which the interaction between Pyk2 and cortactin was examined. **A.** Equal amounts of purified cortactin conjugated to AminoLink beads were incubated with increasing amounts of purified Pyk2. Bound protein was separated on SDS-PAGE gel, gel was stained using Coomassie blue-silver, de-stained, and scanned. BSA that leaked off the beads during the reaction is used here as a loading control. **B.** The scanned image was inverted using ImageJ, and density of individual bands (selected by drawing a box, see section 5.1.3) and background was quantified. **C.** Example of density quantification of Pyk2 bands including subtraction of background density (from lane 1). **D.** An example of a graph of Pyk2 signal against concentration of Pyk2 bound from the same experiment. Note that this pull-down assay yields sufficient points around the *K*_d_ and is fully saturated. **E.** Calculation of dissociation constant (*K*_d_), standard error, confidence intervals, and R^2^ values using GraphPad Prism. **F.** An example of a graph and calculated dissociation constant from three independent repeats of the experiment, where integrated density data was converted to “fraction bound”.

1. Preparation of bait-conjugated beads
 This part describes the preparation of the beads carrying the bait protein to be used in the quantitative pull-down assay. We routinely use AminoLink beads, although the assay could be performed using other types of beads such as glutathione sepharose or Ni-NTA agarose for tagged proteins. The advantage of AminoLink beads is that the protein of interest is covalently attached to the beads; we found that there is limited to no leakage of the protein from the beads after efficient coupling. This also leads to less batch-to-batch variation of the prepared beads. To avoid non-specific binding of the prey protein during the pull-down assay, the remaining active sites on the beads need to be blocked following coupling to the bait protein (**Fig. 2**).
  1.1. Place 0.5 ml of AminoLink bead slurry (50% beads-50% buffer) in an Eppendorf tube. Keep careful track of the amount of slurry used to be able to reconstitute this ratio later.
  1.2. Wash three times with coupling buffer.
 **TIP**: The volume of wash buffer during the washing steps is not important. Use a large volume of the buffer here to ensure complete washing.
  1.3. Buffer exchange purified recombinant bait protein into coupling buffer.
 **NOTE**: Buffers should not contain any primary amines, as these will couple to AminoLink beads and interfere with binding efficiency.
  1.4. Spin protein at 20000 g, 4°C in a tabletop centrifuge to test protein solubility. Determine protein concentration with a colorimetric assay (*i.e.* Bradford dye) or by NanoDrop.
  1.5. Add purified recombinant bait protein to the washed AminoLink beads. The protein concentration bound to 10 μl of bead slurry should be below the anticipated *K*_d_ in a final reaction volume of 500 μl. Save a small amount for subsequent determination of coupling efficiency. Example: to determine the binding affinity of cortactin to Pyk2, we conducted a literature search to obtain values for cortactin binding to other proteins such as Arg kinase [5]. From this search we anticipated a *K*_d_ around 0.5 μM. Cortactin has a molecular weight of 61.5 kDa. To obtain a cortactin concentration of 0.1 μM in 500 μl reaction volume, we coupled 3.1 μg of cortactin to 10 μl bead slurry. We used 155 μg cortactin for 0.5 ml of beads.
 **NOTE**: In the case where no *K*_d_ information could be found in the literature, we recommend to start with a concentration of bait protein between 0.1 and 1 μM.
  1.6. Add 5M sodium cyanoborohydride (20 μl/ml) to the reaction slurry (in a chemical hood).
  1.7. Rotate end-over-end overnight at 4°C.
  1.8. Spin at 8000 g, 4°C for 2 min in a tabletop centrifuge, remove supernatant. Determine binding efficiency by comparing the absorbance of supernatant to input. Binding efficiency should be close to 100%.
  1.9. Wash beads three times with quenching buffer.
  1.10. To block the remaining active sites on the beads, add 1 ml quenching buffer containing 100 mg/ml BSA, and 5M sodium cyanoborohydride (20 μl/ml).
 **TIP**: BSA will leak off the beads in subsequent binding assays due to the high concentration used in blocking. This may interfere with the assay if the molecular weight of the prey protein is similar to the molecular weight of BSA. In such cases, beads can be blocked with 1 ml of 50 mM ethanolamine in PBS instead of BSA.
  1.11. Block beads overnight (or for a minimum of 6 to 8 h).
  1.12. Centrifuge at 8000 g, 4°C and remove buffer by aspiration. Take care not to aspirate any beads.
  1.13. Wash at least four times with wash solution and monitor for complete removal of uncoupled BSA with Bradford dye. Wash until no protein is detected in the wash solution. Add binding buffer to make a 50%-50% ratio of bead to buffer slurry.
 **NOTE**: Beads can be stored at 4°C for up to one week. If longer storage time is desired, replace the buffer with 2 × binding buffer containing 50% glycerol and store at −80°C. Remember to keep the bead slurry at a ratio of 50% beads to 50% buffer.
  1.14. To make BSA control beads, start at the blocking step (step 1.10) using AminoLink beads that were washed with blocking buffer.
 **HINT**: Carefully consider which protein to bind to the beads and which protein to keep in solution. The protein bound to the beads is required at much lower concentration compared to the protein in solution. It can also be kept at higher salt concentrations prior to binding to the beads. As a general rule, the protein that is harder to work with is coupled to the beads.
2. Preparation of prey protein solution
  2.1. Purify the desired protein according to your standard protocol.
  2.2. Buffer exchange the protein into binding buffer.
 **TIP**: Buffer conditions may be adjusted to facilitate protein solubility. However, the buffer should be as close as possible to physiological salt conditions and should contain mild detergents only.
  2.3. Concentrate the prey protein using an Amicon centrifugal filter with appropriate molecular weight cut-off.
 **NOTE**: It is important to obtain the prey protein in high enough concentrations to saturate the binding isotherm without introducing reagents that may interfere with binding.
  2.4. Determine if the protein is stable under these conditions.
    2.4.1. Spin the protein at 20000 g, 4°C for 10 min. Visually inspect the tube for the presence of a large protein pellet.
    2.4.2. Pre-clear the protein by adding 20 μl BSA beads per each ml of protein sample. Rotate end- over-end for 30 min at 4°C.
  2.5. Spin down the beads for five minutes at 14000 g, 4°C. Carefully remove and keep the supernatant containing the protein in solution.
  2.6. Determine protein concentrations before and after steps 2.4.1 and 2.5. If a significant amount of protein is lost, adjust buffer conditions to stabilize the protein [6].
3. Screening for binding conditions
 Before beginning the actual binding experiments, it is important to ensure that the protein in solution will bind specifically to the protein beads, but not to the BSA control beads (**Fig. 3**).
  3.1. Prepare four dilutions of the protein in solution (in a total volume of 500 μl each). Highest concentration should be approximately five times the expected *K*_d_. The lowest concentrations should be about 0.5 × the expected *K*_d_. Add 10 μl of bead slurry containing the bait protein.
 **TIP**: The concentration of the bait protein is held constant during the experiment (by using a constant amount of beads, but should be below the anticipated *K*_d_).
  3.2. Repeat this process with 10 μl of BSA control beads instead of protein beads.
  3.3. Rotate end-over-end at 4°C for one hour.
  3.4. Spin down beads for two minutes at 8000 g, 4°C.
  3.5. Carefully remove supernatant and transfer to a fresh tube.
  3.6. Wash twice quickly with binding buffer.
 **TIP**: In order to avoid the loss of beads, it is not necessary to remove the entire buffer in the first two wash steps. To avoid disturbing the binding equilibrium, wash steps should be performed as quickly as possible with a small volume of buffer containing 5%–10% glycerol [3].
  3.7. After the last wash, aspirate the entire wash buffer without disturbing the beads. Use a 27G × 1/2 needle attached to 1 ml syringe to aspirate the remaining traces of buffer.
  3.8. Add 40 μl of LSB and boil samples for 10 min. Alternatively, samples can be incubated on a heat block at 95°C for 10 min. This step will disrupt the binding between bait and prey protein. The bait protein will remain covalently bound to the beads, whereas the prey protein will be released to the supernatant.
  3.9. Spin samples for 5 min at 20000 g in a tabletop centrifuge to precipitate bait-conjugated beads.
  3.10. Load 35 μl of sample per well on an SDS-PAGE gel. Load supernatant only. Add the same volume in each lane and avoid loading any beads. Run the gel and stain with Coomassie blue-silver overnight.
  3.11. De-stain gels completely by washing several times with DDW until no dye comes out of the gel. This may take several hours. Prey protein should be visible in samples pulled-down by bait protein beads, but not by BSA control beads (**Fig. 3B** and **3C**).
 **TROUBLESHOOTING:** Very little protein should be pulled-down with the BSA control beads. If a similar amount of protein is pulled-down with BSA control beads compared to bait-conjugated beads, adjust buffer conditions (see section 2.6).
4. Performing the binding assay
 Quantitative pull-down assay using purified recombinant proteins provides a simple method to obtain the dissociation constant (*K*_d_). The value of *K*_d_ often determines the affinity of binding between two proteins. The dissociation constant is defined by the following formula:
 *K*_d_ = k^-^/k^+^ = (A_eq_)(B_eq_)/(AB_eq_)
 k^-^ = dissociation rate constant (expressed in s^-1^); the probability that the complex will dissociate in a unit of time.
 k^+^ = association rate constant (expressed in M^-1^s^-1^); the probability that two molecules at specific free concentrations will interact at a given moment of time.
 A_eq_ and B_eq_ = concentrations of the free reactants at equilibrium.
 AB_eq_ = concentration of product/complex at equilibrium.
 From the formula above it could be concluded that the lower the *K*_d_, the stronger the interaction, and the more completely A and B are converted into the product AB.
 Two important conditions must be met in order to calculate a proper dissociation constant from the binding curve: First, the graph must contain most of the data points on the slope of the graph close to the *K*_d_. Second the binding curve must reach saturation. The researcher must therefore empirically determine which protein concentrations to use. It is helpful to determine an expected *K*_d_ by searching the literature for similar protein-protein interactions. Ideally, maximal protein concentration should be about 5–10 times the *K*_d_.
  4.1. Make serial dilutions of the prey protein. The highest concentration should be about 5–10 times the expected *K*_d_, with two to three points below the expected *K*_d_. Concentrations will range from 0 μM to five to ten times the expected *K*_d_, nine samples total, in a volume of 490 μl.
 **EXAMPLE:** The expected *K*_d_ for the cortactin-Pyk2 interaction is approximately 0.5 μM. We therefore selected serial dilutions of Pyk2: 0, 0.16, 0.31, 0.63, 1.25, 2.5, 5.0, 10.0 and 20.0 μM.
  4.2. Add 10 μl of AminoLink beads attached to bait protein, or BSA control beads. Rotate end-over-end for one hour at 4°C.
  4.3. Proceed as in Section 3.3 to 3.9 above. Load samples that were pulled-down with bait protein beads on a different gel from samples pulled-down with BSA beads.
 **TIP**: Carefully pipette supernatant to avoid loading beads on the gel.
  4.4. Stain gels overnight using sufficient amount of Coomassie blue-silver staining solution.
  4.5. De-stain gels completely using DDW until no color comes out of the gels.
5. Gel analysis
  5.1. Analyzing the gels using ImageJ:
    5.1.1. Scan the gels after complete destaining using a gel scanner.
    5.1.2. Open images in ImageJ and invert the images (shift + control + I).
    5.1.3. Draw a box around the region of interest (*i.e.* the highest concentration band).
    5.1.4. Select “analyze - measure” (control + M). A result window containing lane number, area, mean, min, and max values will open. The relevant value is the mean density of the band.
    5.1.5. Move the box to the next band and measure band density in the same way. Repeat this until all band densities are measured (**Fig. 4B**).
 **NOTE**: Include a sample of 0 point in each gel (no prey protein in solution) and subtract its values from the signals obtained. This represents your background signal. If high background signal is obtained with BSA control beads, these should be subtracted from the binding values as well (**Fig. 4C**).
  5.2. Analysis of binding data and determining the dissociation constant using GraphPad Prism:
    5.2.1. For a single binding experiment, select “file—new—new project file”. Select “XY” under “new table and graph”. Under “enter/import data” select: “Y: enter and plot single Y value for each point”. In the spreadsheet, fill in the following data:
 X values: enter the concentrations (in μM) of your prey protein that were used in solution.
 Y values: enter the density values obtained from the gel (**Fig. 4C**).
    5.2.2. On the top toolbar under “analyze” click on the option “fit curve with nonlinear regression”. A window will appear asking you how to analyze the data. Under the “fit” tab select “binding saturation—one site specific binding”.
 **NOTE**: The function of “One site specific binding” calculates the dissociation constant according to the following formula: Y = B_max_ × X / (*K*_d_ + X). Where Y is the specific binding, X is the concentration of the ligand (prey protein), B_max_ is the maximum specific binding in the same units as Y, and *K*_d_ is the dissociation constant, which represents binding affinity. The *K*_d_ value is calculated by extrapolating the prey protein concentration needed to achieve half-maximal binding at equilibrium.
    5.2.3. At the left side of the screen, under “results”, a window of “one site—specific binding” data will appear, as in **Figure 4E**. The first set of values is your actual results (best-fit values). The second set is your standard error (std. error), followed by the 95% confidence intervals. For example, from the table in **Figure 4E**, you can deduce that your *K*_d_ is 0.6317 ± 0.08976 μM. You can say with 95% confidence that the *K*_d_ falls between 0.4194 to 0.8440. The R^2^ value is 0.9839, which indicates a very good curve fit. Look at the graph obtained from your data and convince yourself that you have a good graph with saturated data and sufficient points around the *K*_d_ and at the slope.
 **NOTE**: Density values are arbitrary units, and can vary from gel to gel. Arbitrary values can be changed to percent maximum signal by converting the highest value obtained to 100% and then scaling all other data points accordingly. This can only be done with saturated curves. If your binding curve is not saturated, you cannot assume that the highest data point represents 100% binding.
  5.3. Repeating experiments and combining data: Experiments need to be repeated at least three times in order to combine data and obtain a final *K*_d_ value.
    5.3.1. In GraphPad, select “file—new—new project file”.
    5.3.2. Under “tables and graphs” select “XY”.
    5.3.3. Select “enter/import data” and enter 3 replicate values in side-by-side sub-columns. Perform the analysis as described in 5.2 above for the single experiments. You will now get a graph with combined data and error bars (**Fig. 4F**).
 **NOTE**: For additional reading, use the following link: http://www.graphpad.com/guides/prism/6/curve-fitting/.

## ANTICIPATED RESULTS

The study of protein-protein interactions is a key component of understanding signaling pathways. Despite the importance of these interactions, very few studies are quantitative or report binding affinities. Quantitative pull-downs with purified recombinant proteins provide a simple method to obtain dissociation constants (*K*_d_). These quantifications are invaluable to compare relative binding of proteins, to map binding sites, and to show that binding is direct.

The method described in this paper provides a guideline for planning and execution of efficient quantitative binding assays. It requires that one protein, the prey protein, is soluble in high concentrations at close to physiological salt conditions. The protein bound to the beads (the bait protein) is required at lesser concentrations and can be kept in higher salt buffers. The first important step is thus selecting which protein to bind to the beads. In general, the protein that is more difficult to purify, concentrate, or to keep in solution should be bound to the beads. Careful determination of solubility for both proteins before the screen is vital and will save a lot of work later in the process [6].

An initial screening experiment will show if the prey protein binds specifically to the bait protein beads, and not to the BSA control beads. To avoid staining differences between different gels, it is advisable to run this experiment side by side on the same gel (**Fig. 3**). This preliminary experiment will not provide enough data points to calculate a *K*_d_, however, it will show if the chosen buffer conditions are appropriate for binding and that the binding is specific. Including this step will save proteins and other valuable reagents.

We found that the removal of potentially unfolded protein by centrifugation (Step 2.4.1) and pre-clearing with control beads (Step 2.4.2) significantly reduces non-specific binding during the assay itself. We also designed this experiment to be done with a minimal amount of beads to avoid nonspecific bead trapping. Minimal binding to control beads at higher concentrations is acceptable and can be adjusted for. If available, a prey protein known to bind to the protein on beads may be included as a positive control.

For the assay itself, bait protein beads are incubated with increasing concentrations of prey protein. Bound prey protein is then separated on SDS PAGE gel, which is stained with Coomassie blue silver. The blue silver staining method is highly sensitive to lower protein concentrations (detection limit of 1 ng protein) and highly reproducible due to its insensitivity to a number of parameters such as temperature, quality of solvents, and developing time [4].

For data analysis, the de-stained gel is scanned and imported into suitable analysis software. If ImageJ is used, the image must be inverted prior to analysis. Using this method, we measured the integrated density of the pulled-down protein in each lane and performed a background subtraction by manually subtracting the zero input lane. Plotting prey protein concentrations versus integrated density will yield a binding curve that should be hyperbolic (**Fig. 4D**). If the curve is linear, binding is not saturated and the assay must be repeated with higher prey protein concentrations.

Several trials may be required to determine which concentrations of prey protein will yield a valid binding curve. At the lower end, enough data points must be obtained around the *K*_d_, at the slope of the binding hyperbola. Most data analysis softwares will provide a *K*_d_ for curves with insufficient data points at the slope, however, these results are not valid. It is equally important to achieve binding saturation. Without saturation, a *K*_d_ cannot be calculated. Once appropriate protein concentrations are determined the assay should be repeated at least three times. To combine the data, we converted the measured from integrated density (in arbitrary units) to fraction bound. This calculation sets binding at saturation to 100% and can therefore only be performed when binding is saturated (**Fig. 4F**).

In general, a dissociation constant of a binding reaction should be measured in equilibrium conditions. A typical pull-down assay prepares an equilibrium mixture of reactants and products, however by precipitating the complex and washing the complex-bound beads the prey protein may start to dissociate into solution in order to achieve a new equilibrium. A simple way that was suggested to avoid alteration of the equilibrium is to measure the concentration of the fraction of prey protein that did not bind to the bait protein on the beads and to calculate indirectly the concentration of bait-bound prey protein on the beads by subtraction [3]. While this is a good suggestion in theory, it would be practically difficult to apply that to our assay. First, performing the assay without washing the beads assumes that all prey protein excluded from the supernatant binds specifically to the bait protein on the beads and not to the beads themselves in a non-specific manner. Although the bead blocking stage is usually efficient, prey protein will still bind to the beads non-specifically during the spin and needs to be removed. Alternatively, one can try and subtract the signal of control BSA beads from the pulled down prey protein signal. Second, depletion of the protein from the supernatant at low concentrations cannot be measured in practice because the solutions are too dilute and the assays have to be performed in a relatively large solution volume (minimum 500 μl). If a subtraction assay is necessary instead of direct pull-down assay, one could try and solve this problem by western blotting, which would be a more sensitive method for detection of the specific prey protein.

A modification of the *in vitro* binding assay could be used for measuring competitive inhibition of protein-protein interactions. Such assays could be applied for drug discovery of protein-protein interaction inhibitors. Theoretically, it is possible to add a constant concentration of a competitive inhibitor to the *in vitro* binding assay. In such a case, the *K*_d_ should increase when compared to the original experiment (*K*_d_ app). The inhibition constant K_i_ could then be calculated by using the following equation: *K*_d_ app = Km (1+ [I]/K_i_).

Once validated *in vitro*, the biological relevance and significance of any protein-protein interaction should be examined in its physiological context within the cell. An *in vitro* pull-down assay could lead to false positive results if, for example, it shows binding between two proteins that are not naturally localized to the same place in the cell. It is therefore necessary to back up the *in vitro* pull-down studies with other experimental techniques such as co-localization, fluorescence recovery after photobleaching (FRET), or co-immunoprecipitation.

## TROUBLESHOOTING

Potential problems and troubleshooting suggestions are listed in **Table 1**.

**Table 1.** Troubleshooting table.

Step	Problem	Causes	Suggestions
1.8	Low coupling efficiency of bait protein to beads	Sample buffer contains primary amines	• Avoid using buffers or reagents containing primary amines during initial protein purification • Remove any primary amines by buffer exchange
2.2, 2.4	Prey protein not soluble at high concentrations in binding buffer	Buffer conditions not compatible with protein	• Carefully screen protein solubility (adjust salt, pH, detergents) [6]. • Couple this protein to the beads
2.3	Protein precipitates during concentration with Amicon centrifugal filters	• Protein solubility • Spin to harsh • High local concentrations of protein on bottom of the filter	• See step 2.2 • Pre-soak filters in buffer containing 50% glycerol • Reduce centrifugation speed • Centrifuge in five minute increments, pipette protein sample up and down between spins
3.11	Prey protein binds to BSA beads	Protein is not stable	• Screen buffer conditions [6] • Ensure that beads are completely blocked • Block with ethanolamine instead of BSA
3.11	Prey protein is degraded	Protein is not stable	• Run gel of input to determine if the protein was degraded prior to assay • Shorten incubation time • Screen buffer conditions
3.11	Variation in *K*_d_ between experimental repeats	Binding efficiency varies due to temperature and buffer conditions	• Conduct all binding experiments at a constant temperature of 4°C • Use the same buffer for all repeats • Perform all experiments with the same buffer, especially when comparing the binding constants of wild-type versus mutant protein
3.11, 4.5, 5.2	Binding does not increase with increased protein concentration	Binding may be saturated	• Lower prey protein concentrations
3.11, 4.5, 5.2	Binding pattern erratic	Bead volume not even between different reaction tubes	• Carefully pipette equal amounts of beads • Slightly cut or trim pipette tips prior to pipetting beads
3.11, 4.3	SDS-PAGE gels expand, uneven running of gels	Beads loaded on gel	• Carefully avoid loading beads • Increase centrifugation time/speed prior to loading
3.11	No binding to bait protein beads	Concentration of bait or prey protein is too low	• Increase concentration of prey protein • Bind more bait. protein to beads
3.11	Bait protein does not bind	No interactions between bait and prey protein	• Check protein beads with a prey protein that is known to bind to bait (positive control)

## Abbreviations used

BSA: bovine serum albumin
DDW: double distilled water
FRET: fluorescence recovery after photobleaching
*K*_d_: dissociation constant
kDa: kiloDalton
LSB: lamelli sample buffer
mg: milligram
mM: milimolar
MW: molecular weight
PBS: phosphate buffered saline
Pyk2: proline-rich tyrosine kinase 2
SDS-PAGE: sodium dodecyl sulfate-polyacrylamide gel electrophoresis
